# Reduced Transforming Growth Factor-β Activity in the Endometrium of Women With Heavy Menstrual Bleeding

**DOI:** 10.1210/jc.2016-3437

**Published:** 2017-01-03

**Authors:** Jacqueline A. Maybin, Lyndsey Boswell, Vicky J. Young, William C. Duncan, Hilary O. D. Critchley

**Affiliations:** Medical Research Council Centre for Reproductive Health, University of Edinburgh, Queen’s Medical Research Institute, Edinburgh EH16 4TJ, United Kingdom

## Abstract

**Context**:**

Heavy menstrual bleeding (HMB) is common and incapacitating. Aberrant menstrual endometrial repair may result in HMB. The transforming growth factor (TGF)-*β* superfamily contributes to tissue repair, but its role in HMB is unknown.

**Objective:**

We hypothesized that TGF-*β*1 is important for endometrial repair, and women with HMB have aberrant TGF-*β*1 activity at menses.

**Participants/Setting:**

Endometrial biopsies were collected from women, and menstrual blood loss objectively measured [HMB >80 mL/cycle; normal menstrual bleeding (NMB) <80 mL].

**Design:**

Immunohistochemistry and reverse transcription polymerase chain reaction examined endometrial TGF-*β*1 ligand, receptors, and downstream SMADs in women with NMB and HMB. The function and regulation of TGF-*β*1 were examined using cell culture.

**Results:**

*TGFB1* mRNA was maximal immediately prior to menses, but no differences detected between women with NMB and HMB at any cycle stage. Histoscoring of TGFB1 revealed reduced staining in the stroma during menses in women with HMB (*P* < 0.05). There were no significant differences in *TGFBR1/2* or TGFBR1/2 immunostaining. Cortisol increased activation of TGFB1 in the supernatant of human endometrial stromal cells (HES; *P* < 0.05) via thrombospondin-1. Endometrial *SMAD2* and *SMAD3* were lower in women with HMB during menstruation (*P* < 0.05), and decreased phosphorylated SMAD2/3 immunostaining was seen in glandular epithelial cells during the late secretory phase (*P* < 0.05). Wound scratch assays revealed increased repair in HES cells treated with TGF-*β*1 *versus* control (*P* < 0.05).

**Conclusions:**

Women with HMB had decreased TGF-*β*1 and SMADs perimenstrually. Cortisol activated latent TGF-*β*1 to enhance endometrial stromal cell repair. Decreased TGF-*β*1 activity may hinder repair of the denuded menstrual endometrium, resulting in HMB.

## Teaser

TGF-*β*1 and downstream SMADs were decreased in perimenstrual endometrium from women with heavy menstruation *versus* controls. TGF-*β*1 was activated by cortisol in endometrial cells and enhanced repair.

The human endometrium is a complex and dynamic tissue. Throughout the reproductive years of a woman’s life, it responds to steroid hormones to prepare for implantation, shed its luminal portion in the absence of pregnancy, and efficiently regenerate for the subsequent menstrual cycle. Menstruation occurs as a result of the sharp ecline in progesterone as the corpus luteum regresses. This progesterone withdrawal stimulates an influx of inflammatory cells and release of matrix metalloproteinases, resulting in tissue destruction and menstrual bleeding (1, 2).

The regulation of endometrial repair after shedding remains undefined. Scanning electron microscopy and hysteroscopy analysis revealed that luminal epithelial cell migration precedes stromal expansion, but that breakdown and repair occur simultaneously in adjacent sections of the human endometrium during active bleeding (3). Initiation of endometrial repair therefore occurs during the menstrual phase, when ovarian hormone levels remain low. Indeed, in the mouse model of simulated menstruation, repair occurred without delay when both exogenous and endogenous estrogens were removed (4).

The transforming growth factor (TGF)-*β* superfamily includes TGF-*β*s, activins, and nodal and bone morphogenic proteins. This superfamily has been implicated in cell motility, proliferation, apoptosis, immune response, and differentiation [reviewed in (5)]. Therefore, they are attractive candidates for the coordination of endometrial repair at menses.

TGF-*β* is synthesized as a dimeric preproprotein and is released in a latent form. It is activated in a tissue-specific fashion by a variety of mechanisms, including extremes of pH or via plasmin or thrombospondin-1 (TSP-1) (6, 7). Once activated, it binds to type II transmembrane serine/threonine kinase receptors, which then form a heterotetrameric complex with dimers of type I receptors. This leads to phosphorylation and activation of intracellular regulatory SMADs (SMAD2 and 3), which in turn interact with the comediator SMAD4 and translocate to the nucleus to regulate transcription of target genes. TGF-*β* ligands and receptors are present in the human endometrium with maximal levels found during menstruation (8). TGF-*β* ligand expression was found to be suppressed by progesterone (8), meaning endometrial induction following progesterone withdrawal is expected. Despite the low levels of circulating progesterone and estradiol at menses, local generation of steroids in the endometrium may play a vital role in menstrual physiology. Endometrial expression of the enzyme 11*β*HSD1, necessary for local generation of cortisol, and the expression of the glucocorticoid receptor have both been reported to be upregulated at the time of menses (9). The role of cortisol in the regulation of TGF-*β* remains undetermined.

Heavy menstrual bleeding (HMB) of endometrial origin (HMB-E) is a common condition with a significant impact on the quality of life of otherwise healthy women (10). The financial costs to women, their families, and employers are marked (11). HMB-E can be contributed, at least in part, to delayed or ineffective endometrial repair at menses. Identification of the mechanisms involved in endometrial repair and aberrations in women with HMB-E will lead to new, effective medical therapies for the many women suffering from this debilitating condition.

In this study, we hypothesize that TGF-*β*1, its receptors, and downstream SMADs are important for endometrial repair at menses, and that women with HMB-E have aberrant expression of this superfamily prior to and during the menstrual phase. To investigate this, we used well-categorized endometrial whole tissue biopsies from women with objectively measured normal (<80 mL) and heavy (>80 mL) menstrual blood loss alongside *in vitro* endometrial cell culture and functional assays.

## Materials and Methods

### Tissue collection

Endometrial biopsies were collected with an endometrial suction curette (Pipelle, Laboratorie CCD, Paris, France) from 91 healthy women of reproductive age, who were predominantly White/Caucasian. Written informed consent was obtained, and ethical approval was granted from Lothian Research Ethics Committee (LREC/07/S1103/29). Participants were aged 22 to 50 years (median 41; mean 41). All reported regular menstrual cycles (21 to 35 days) and had not taken any exogenous hormones or used an intrauterine device for 3 months prior to tissue collection. Women with large fibroids (>3 cm) and endometriosis were excluded.

Immediately after collection, tissue was divided when possible and placed in the following: (1) RNA later stabilization solution [Ambion (Europe), Warrington, UK] and stored at −70°C for RNA extraction; (2) neutral buffered formalin prior to paraffin wax embedding; and (3) phosphate-buffered saline (PBS) for stromal cell extraction. If limited tissue was obtained (which often occurred with menstrual phase collection), neutral buffered formalin fixation was prioritized.

Menstrual stage was carefully categorized according to the following: (1) histological appearance based on the criteria of Noyes *et al.* (12), assessed by a consultant pathologist; (2) the participant’s reported last menstrual period; and (3) serum progesterone and estradiol levels at the time of biopsy (see Supplemental Methods). Consistency for all 3 parameters was necessary before inclusion. Six endometrial tissue samples were excluded due to inconsistent dating and 1 sample due to detection of hyperplasia. Biopsies were classified as proliferative, early-mid secretory, late secretory, or menstrual for analysis (Supplemental Table 1).

### Objective menstrual blood loss measurement

A subset of women (n = 78) also had objective measurement of their menstrual blood loss using the modified alkaline hematin method, as previously published (13, 14). In brief, women were given the same brand of tampon and/or pad (Tampax tampons and Always towels, Proctor and Gamble, Weybridge, UK), with verbal and written instruction on collection. Used sanitary products were added to a measured volume of 5% sodium hydroxide. The contents were left for 24 hours to allow conversion of hemoglobin to hematin. During the same time period, a 1 in 200 dilution of the patient’s venous blood in 5% sodium hydroxide was made and stored separately. The optical density (OD) of the samples was then measured using spectrophotometry at 546 nm (*A*_546_). Menstrual blood loss (MBL) was calculated using the following equation (13):

$$\text{MBL}=\frac{\left(\text{OD}\;\text{of}\;\text{menstrual}\;\text{blood}\;\text{solution}\times \text{total}\;\text{volume}\;\text{of}\;\text{added}\;\text{NaOH}\right)}{\left(\text{OD}\;\text{of}\;\text{venous}\;\text{blood}\times 200\right)}$$

Greater than 80 mL was classified as HMB, and <80 mL as normal (NMB).

### Immunohistochemistry

The 5-µm tissue sections were deparaffinized in xylene and rehydrated. Slides for TGF-*β*RI and II were loaded into a Celerus Riptide decloaking chamber (Celerus Diagnostics, Carpinteria, CA). Epitope retrieval was performed using Novocastra Epitope Retrieval solution Ph6 (Leica Microsystems, Ernst-Leitz-Straße, Wetzlar, Germany). Slides were loaded onto Leica Bond-Max automated immunostainer (Leica Microsystems). Primary antibodies were applied for 2 hours at 37°C (see Supplemental Table 2), and negative control tissues were incubated with isotype-matched IgG at the same concentration as the primary antibody. The presence of antigen was visualized with Bond Polymer refine detection kit (Leica Microsystems). TGF-*β*1 detection was performed on the laboratory bench after pH9 antigen retrieval. The ImmPRESS polymerized reporter system (Vector Laboratories, Peterborough, UK) was used before liquid diaminobenzidine kit (Zymed Laboratories, San Francisco, CA) detection. Sections were counterstained with hematoxylin, dehydrated, and mounted with Pertex (Cellpath, Hemel Hempstead, UK).

### Semiquantitive histoscoring

Localization and intensity of immunostaining were evaluated by two independent, masked observers (15). The intensity of staining was graded with a 3-point scale (0 = no staining, 1 = mild staining, 2 = strong staining). This was applied to the glands and stromal cells, as well as the surface epithelium and endothelial cells where visualized (note: the latter two cellular components were often absent in menstrual phase tissue, accounting for the lower n numbers in these groups). The percentage of tissue in each intensity scale was recorded (15). A value was derived for each of the cellular compartments by using the sum of these percentages after multiplication by the intensity of staining.

### Cell culture

Primary human endometrial stromal (HES) cells were isolated from secretory endometrial tissue (n = 6) by enzymatic digestion, as previously described (16). These women met the criteria detailed previously but did not undergo objective measurement of their menstrual blood loss. Cells were cultured in RPMI 1640 medium supplemented with 10% fetal calf serum, 1% 200 mM L-glutamine, and 500 mg/mL gentamycin.

Secretory phase HES cells from 3 patients with a subjective complaint of HMB and not using oral or inhaled corticosteroids were plated at 3 × 10^5^ cells/well in 6-well plates in 10% RPMI 1640. The next day, cells were washed in PBS and incubated in serum-free media overnight. Cells were then treated for 24 hours in duplicate with the following: (1) vehicle (1:1000 absolute ethanol); (2) 1 µM cortisol (17); or (3) 1 μM cortisol plus 5 μM leucine-serine-lysine-leucine (LSKL), a TSP-1 inhibitor (following a 2-hour pretreatment with 5 μM LSKL alone). The cell supernatant was collected for enzyme-linked immunosorbent assay (ELISA), and RNA was extracted from cells.

### Quantitative reverse transcription polymerase chain reaction

Total RNA from cells and endometrial biopsies was extracted using the RNeasy Mini Kit (Qiagen, Sussex, UK) with on-column DNase I digestion, according to manufacturer’s instructions. RNA samples were reverse transcribed using the Superscript VILO cDNA synthesis kit (Invitrogen, Paisley, UK), according to manufacturer’s instruction, with appropriate controls. Primers for each gene of interest were designed using the Universal Probe Library Assay Design Center (Roche Applied Science, Burgess Hill, UK) (see Supplemental Table 3) and purchased from Eurofins (MGW Operon, Ebergsberg, Germany). Polymerase chain reaction was carried out using ABI Prism 7900 (Thermo Fisher Scientific, Loughborough, UK). Samples and controls were analyzed in triplicate using Sequence Detector version 2.3 (Thermo Fisher Scientific), using the comparative threshold method. Messenger RNA (mRNA) transcripts were normalized relative to the geomean of two appropriate housekeeping genes, 18S and ATP5B, as determined by geNorm assay (Primerdesign, Southampton, UK), and quantified relative to a positive human liver cDNA sample.

### ELISA

A TGF-*β*1 ELISA was performed using a Human TGF-*β*1 Quantikine Kit (DB100B; R&D Systems, Loughborough, UK), according to the manufacturer's instructions. Samples were analyzed without activation and with latent TGF-*β*1 activated to the immunoreactive form using 1 m HCl and neutralized with 1.2 m NaOH/0.5 m HEPES buffer. Samples were assayed in duplicate, and after development assays were measured on a Laboratory Systems Multiscan EX Microplate reader at 450 nm with wavelength correction at 540 nm. Values were determined by standard curve analysis. Intra-assay coefficient of variability was 2.5%, and the between-batch coefficient of variability was 8.3% for cell culture supernatants.

### Wound scratch assay

Secretory phase HES from three participants (passage <5) were seeded at 2 × 10^5^/well in 12-well plates in appropriate supplemented media (see previous discussion), and, 16 hours before scratch, medium was changed to serum free. Each well of cells was scratched with a sterile 200 μL pipette tip, washed with PBS, and then incubated in serum-free media with vehicle, 1 ng human recombinant TGF-*β*1 (PeproTech, London, UK), or 10 μg/mL TGF-*β* type I activin receptor-like kinase receptor inhibitor SB 431542 hydrate (Sigma-Aldridge, Dorset, UK) (n = 3 participants, triplicate wells for each). For each well, 4 to 5 images were captured along the length of each wound at 0 and 24 hours using an Axiovert 200 M inverted microscope (Carl Zeiss, Jena, Germany). Images were analyzed using AxioVision release 4.72, and calculations of average distance closed for each sample were based on three measurements at identical positions along each wound image at 0 and 24 hours.

### Statistical analysis

Analysis was carried out using GraphPad Prism Software (San Diego, CA). For comparison of multiple data sets with two grouping variables (*i.e.*, HMB *versus* NMB and stage of menstrual cycle, mRNA, and immunohistochemistry data), a two-way analysis of variance was used, with Bonferroni’s multiple comparisons test. A paired one-way analysis of variance with Tukey’s multiple comparisons test was used to compare cell culture treatments. Tissue and cell endometrial mRNA results were expressed as the quantity relative to a comparator sample of RNA from human liver. A value of *P* < 0.05 was considered significant.

## Results

### There are increased concentrations of *TGFB1* in the late secretory phase

**Figure 1.**
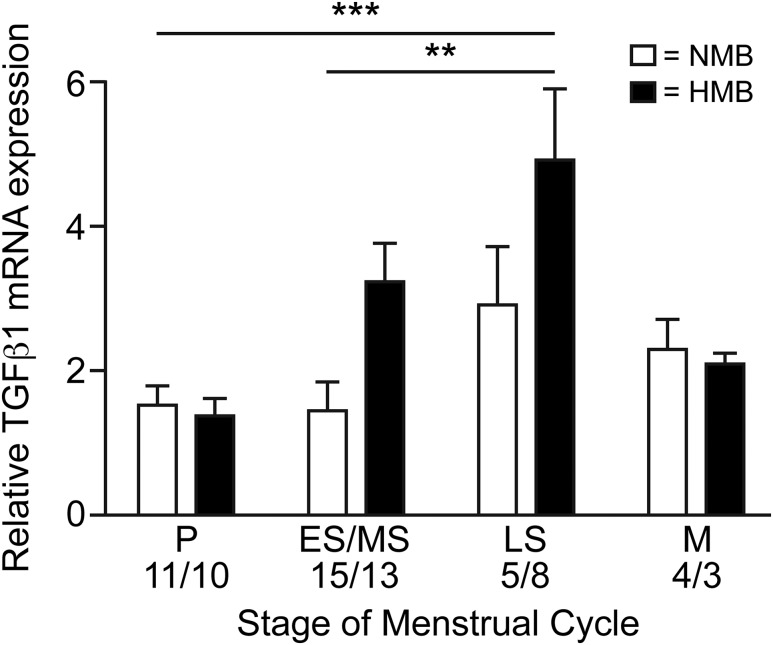
*TGFβ1* in the human endometrium. TGF-*β*1 mRNA concentrations in endometrium from across the menstrual cycle in women with HMB (blood loss >80 mL) and NMB (blood loss <80 mL). E/MS, early-mid secretory; LS, late secretory; M, menstrual; P, proliferative. ***P < 0.001; **P < 0.01.

TGF-*β*1 mRNA was examined by quantitative reverse transcription polymerase chain reaction in whole endometrial biopsies from women sampled at various stages of the menstrual cycle who had objectively determined menstrual blood loss. Overall, the stage of the menstrual cycle had a significant impact on *TGFB1* expression (*P* = 0.0025, *F* = 5.339), with the late secretory phase resulting in significantly higher levels of *TGFB1* than endometrium from the proliferative (*P* < 0.001) or early-mid secretory (*P* < 0.01) phases (Fig. 1). There was no significant difference between the late secretory and menstrual phase. The increased transcription of TGFB1 in the late secretory phase did not continue into the menstrual phase.

### Women with HMB do not have altered endometrial *TGFB1* concentrations or TGF-*β*1 reception

**Figure 2.**
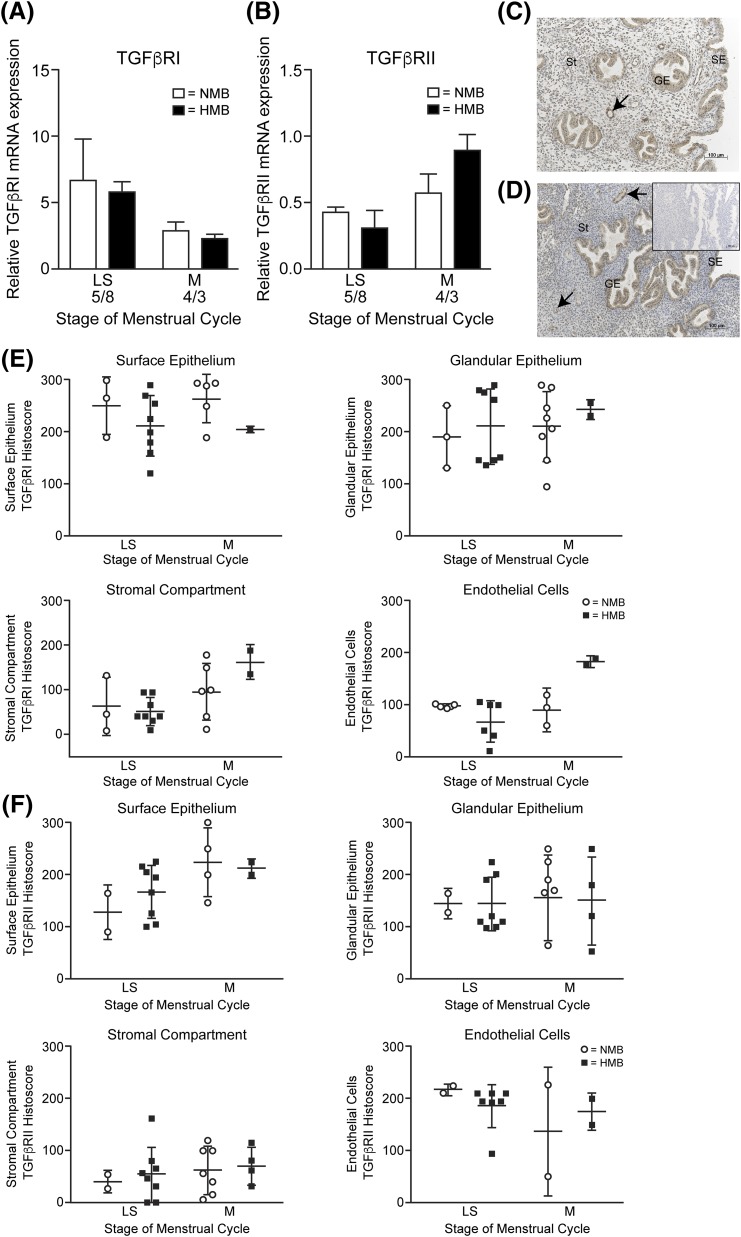
TGF-*β*RI and TGF-*β*RII in the human endometrium before and during menstruation. (A) TGF-*β*RI mRNA concentrations in endometrium from women with normal (NMB; <80 mL) and heavy (HMB; >80 mL) menstrual bleeding during the late secretory (LS) and menstrual (M) phases. (B) TGF-*β*RII mRNA concentrations. (C) Immunohistochemical staining of TGF-*β*RI in endometrium from the late secretory phase. Arrow indicates endothelial cells. (D) Immunohistochemical staining of TGF-*β*RII in endometrium from the late secretory phase; inset: negative control. (E) Immunohistochemical histoscore of TGF-*β*RI in human endometrium from women with heavy and normal bleeding during the late secretory and menstrual phases. (F) Immunohistochemical histoscore of TGF-*β*RII in human endometrium from women with heavy and normal bleeding during the late secretory and menstrual phases. (Note: lower n numbers appear in surface epithelium and endothelial cell scoring due to the inability to identify these cells in some tissues.) GE, glandular epithelium; SE, surface epithelium; St, stromal cell compartment.

We compared the expression of *TGFB1* in women with NMB and HMB (Fig. 1) and found no significant difference in *TGFB1* expression between the two groups at any cycle stage. In addition, the two major TGF*β*1 receptors, type I and type II, were examined in the late secretory and menstrual endometrial samples. Neither *TGFBR1* nor *TGFBR2* expression was significantly different in endometrium from women with HMB *versus* NMB [Fig. 2(A) and 2(B)]. Immunohistochemical staining revealed maximal staining of TGFBR1 in surface and glandular epithelial cells, with lower intensity staining in the stromal compartment [Fig. 2(C)]. TGFBR2 showed a similar pattern, with highest immunostaining in epithelial and endothelial cells [Fig. 2(D)]. Semiquantitative histoscoring by two masked independent observers confirmed no differences in either receptor when comparing women with HMB and NMB throughout the perimenstrual phase [Fig. 2(E) and 2(F)].

### Women with HMB have reduced perimenstrual endometrial stromal TGFB1

**Figure 3.**
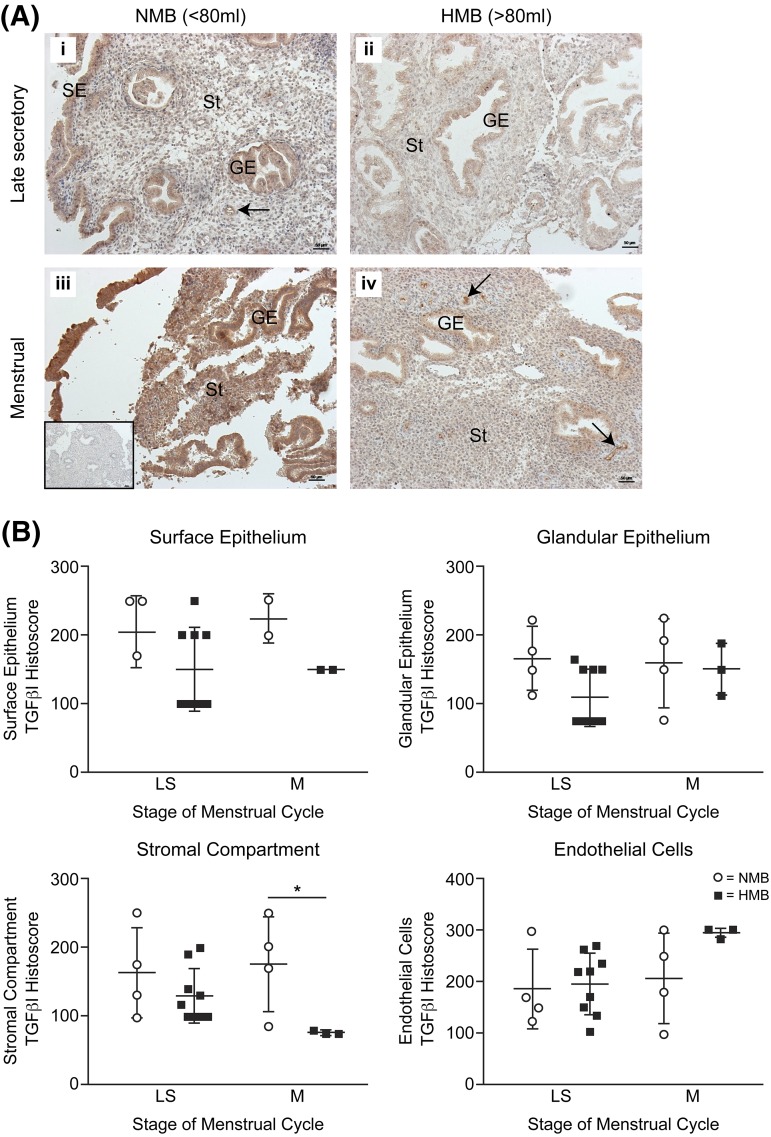
Immunohistochemistry for TGF-*β*1 in human endometrium from the perimenstrual phase. (A) Staining of late secretory (LS) and menstrual (M) phase endometrium from women with HMB (>80 mL) and NMB (<80 mL). Arrows indicate endothelial cells. Inset: negative control. (B) Semiquantitative histoscoring of TGF-*β*1 immunohistochemistry staining. GE, glandular epithelium; SE, surface epithelium; St, stromal compartment. *P < 0.05.

As the numerous cell types in the endometrium expressed TGF-*β*1 receptors, we examined the localization of TGFB1 by immunohistochemistry. TGFB1 could be immunolocalized to the cytoplasm of the surface epithelium, glandular epithelium, stromal cells, and endothelial cells throughout the perimenstrual phase of the cycle in women with NMB (<80 mL) and HMB (>80 mL) [Fig. 3(A)]. Semiquantitative histoscoring revealed that protein in the menstrual phase was similar to late secretory phase. There was significantly reduced TGFB1 staining in the stromal cell compartment of endometrium from women with HMB *versus* those with NMB [Fig. 3(B)]. This suggests some posttranscriptional regulation of TGF-*β*1 in stromal cells.

### Cortisol increases stromal TGF-*β*1 activity via TSP-1

**Figure 4.**
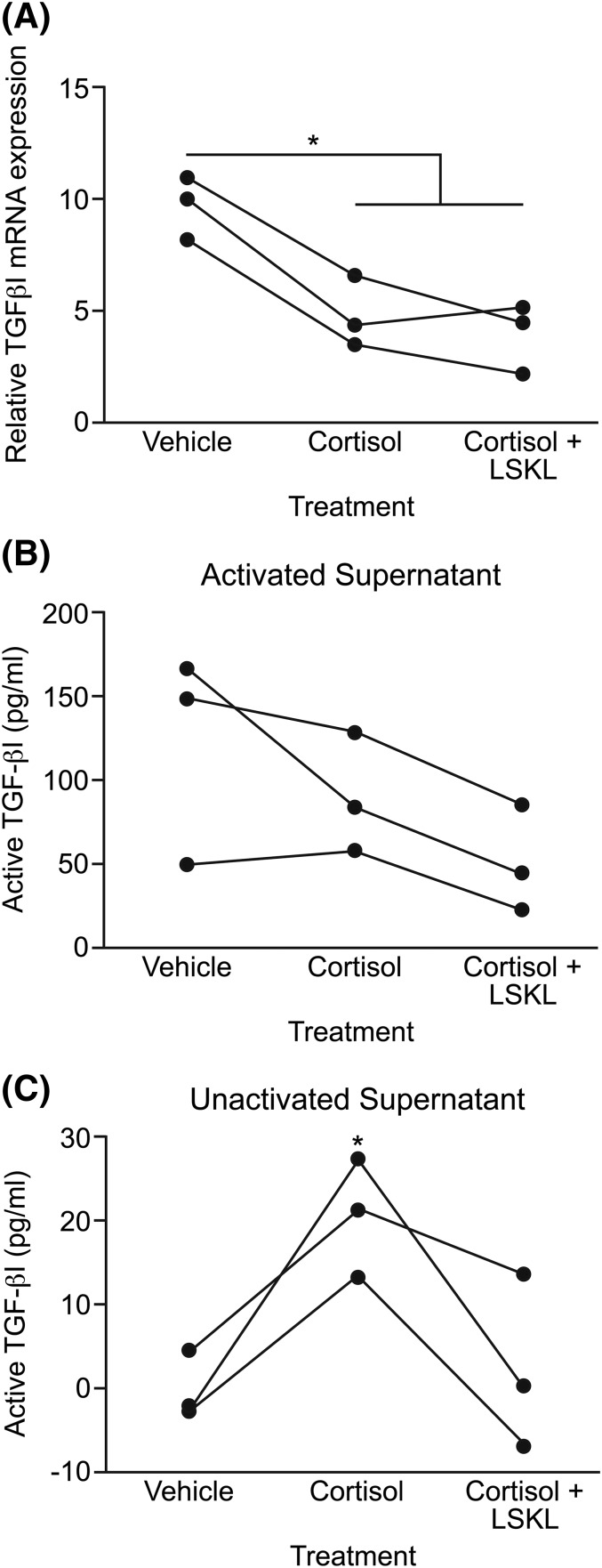
The regulation of TGF-*β*1 by cortisol in primary human endometrial stromal cells. (A) TGF-*β*1 mRNA after 24-hour treatment with vehicle, cortisol (1 μM), or cortisol (1 μM) plus a TSP-1 inhibitor (5 μM LSKL). (B) Active TGF-*β*1 protein levels in experimental culture supernatants following pre-ELISA acid activation of latent TGF-*β*1. (C) Active TGF-*β*1 protein levels in the same culture supernatants without pre-ELISA acid activation (*P < 0.05).

To further investigate the posttranscriptional regulation of TGF-*β*1, we collected primary HES cells from 3 women in the secretory phase of the menstrual cycle for *in vitro* analysis. Perimenstrual serum progesterone and estradiol levels were not significantly different between women with HMB or NMB (Supplemental Table 1). However, we have previously shown that cortisol is involved both in endometrial repair and the regulation of endometrial TSP-1 (14), a known regulator of TGF-*β*1 activity (6). Cortisol or cortisol plus LSKL (a TSP-1 inhibitor) produced a significant decrease in *TGFB1* expression in HES cells [*P* < 0.05, Fig. 4(A)], but there was no difference in the amount of latent TGFB1 secreted, detected by pH activation of culture supernatants prior to detection of activated TGFB1 by ELISA [Fig. 4(B)]. However, analysis of unactivated cell culture supernatants revealed an increase in activation of TGF-*β*1 protein on treatment with cortisol, which was prevented with cotreatment of cells with the TSP-1 inhibitor LSKL [*P* > 0.05, Fig. 4(C)]. These data reveal cortisol does not increase the transcription or latent protein levels of stromal cell TGF-*β*1 but has a role in the activation of latent TGF-*β*1 in human endometrial stromal cells, via TSP-1.

### Women with HMB have reduced perimenstrual endometrial SMAD2/3

**Figure 5.**
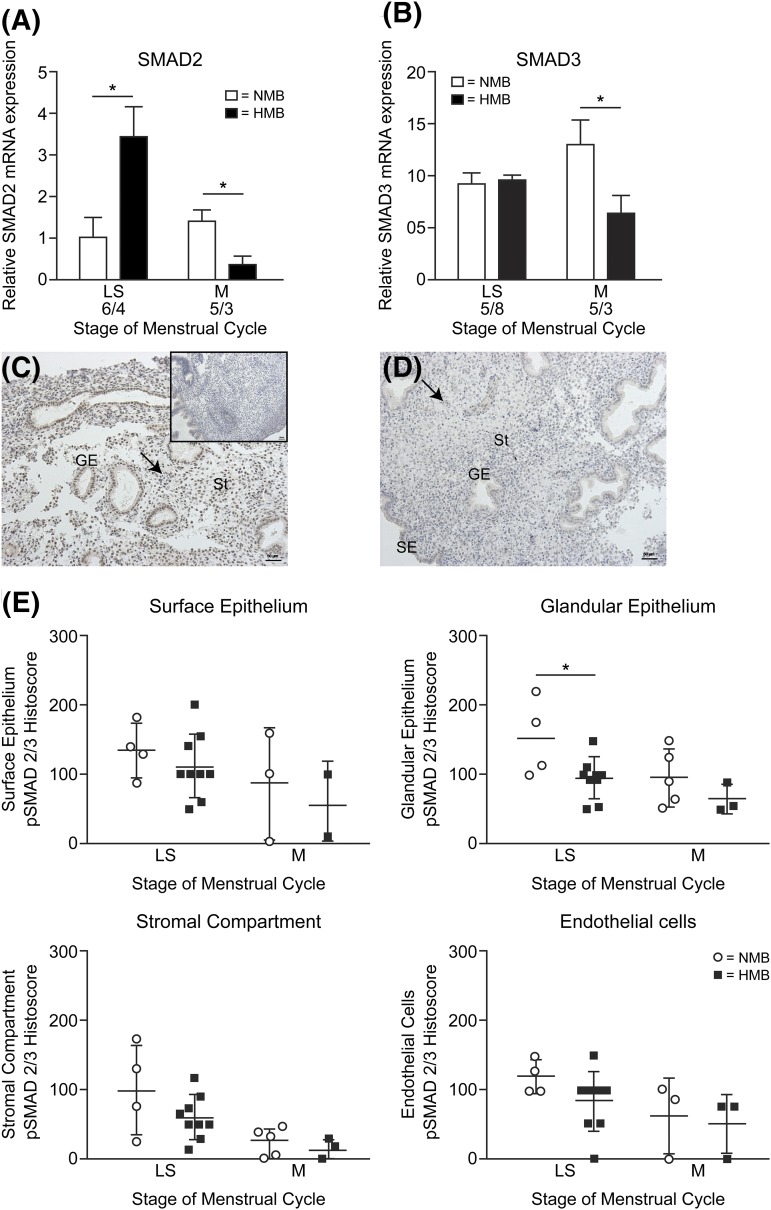
SMAD2/3 in the human endometrium before and during menstruation. (A) SMAD2 mRNA concentrations in endometrium from women with normal (NMB; <80 mL) and heavy (HMB; >80 mL) menstrual bleeding during the late secretory (LS) and menstrual (M) phases. (B) SMAD3 mRNA concentrations in endometrium from women with NMB and HMB in the late secretory and menstrual phases. (C) Phosphorylated SMAD2/3 immunohistochemical staining in late secretory endometrium from a woman with NMB. Inset: negative control. Arrow indicates endothelial cells. (D) Phosphorylated SMAD2/3 immunohistochemical staining in late secretory endometrium from a woman with HMB. (E) Histoscoring of immunostaining for phosphorylated SMAD2/3. GE, glandular epithelium; SE, surface epithelium; St, stromal cell compartment. *P < 0.05.

TGF-*β*1 activity increases the expression and phosphorylation of the regulatory SMADs (SMAD2 and SMAD3). These activated pSMADs then interact with the comediator SMAD4 and translocate to the nucleus to regulate transcription of target genes (5). Examination of *SMAD2* and *SMAD3* expression revealed significant decreases in women with HMB *versus* NMB during the menstrual phase of the cycle [*P* < 0.05, Fig. 5(A) and 5(B)]. SMAD2 was significantly increased in women with HMB *versus* NMB during the late secretory phase [Fig. 5(A)]. Immunohistochemical staining for phosphorylated SMAD2/3 again revealed localization to the glandular epithelium, surface epithelial cells, stromal compartment, and endothelial cells [Fig. 5(C) and 5(D)]. Histoscoring revealed a significant reduction in activated SMAD2/3 protein levels in the endometrial glandular epithelial cells in women with HMB *versus* NMB during the late secretory phase of the menstrual cycle [Fig. 5(D)].

### TGF-*β*1 accelerates wound healing in primary endometrial cells

**Figure 6.**
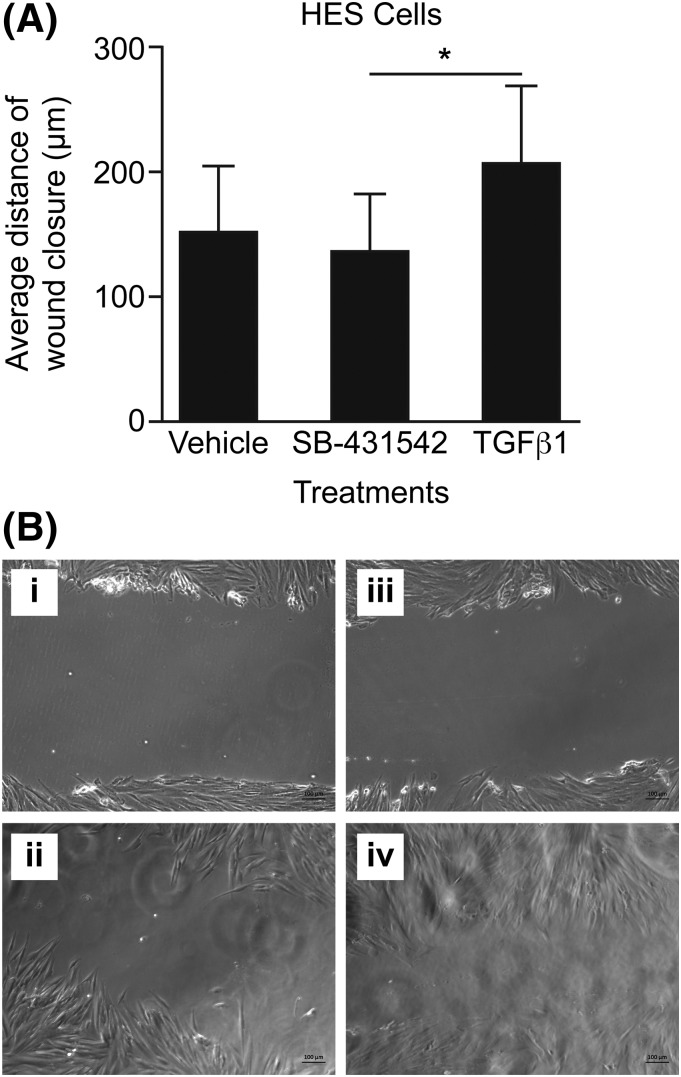
The effect of TGF-*β*1 on human endometrial cell wound repair. (A) Average wound scratch closure distance (scratch distance at 0 hours minus scratch distance at 24 hours) in human primary stromal endometrial cells after treatment with vehicle, the Alk receptor inhibitor SB-431542, or 1 ng TGF-*β*1. (B) Images of wound scratch in HES cells treated with 10 μg/mL SB-431542 for the following: (i) 0 hours; (ii) 24 hours and treated with 1 ng TGF-*β*1; (iii) 0 hours; and (iv) 24 hours. *P < 0.05.

To examine the functional effects of increased TGF-*β*1 activity, primary HES were subjected to a wound scratch assay. As these cells are sources of TGF-*β*1, they were studied in the presence of vehicle, SB-431542 (to block endogenously stimulated phosphorylation of SMAD proteins), or TGF-*β*1. HES cells showed significantly increased wound closure with TGF-*β*1 treatment *versus* SB-431542–treated cells (*P* < 0.05, Fig. 6).

## Discussion

**Figure 7.**
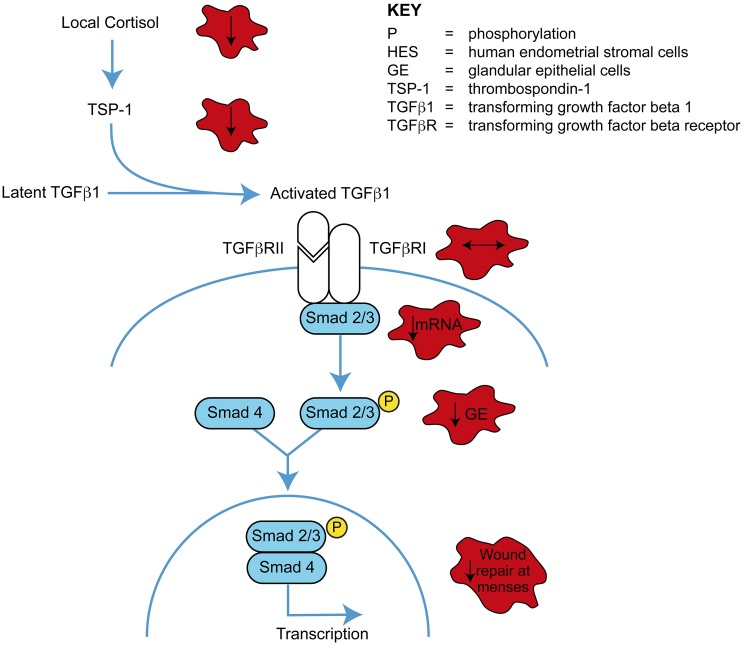
Proposed role of TGF-*β*1 in the human endometrium at menstruation. Red stars represent findings in women with HMB and potential impact on endometrial function.

In this study, we detail significant differences in TGF-*β*1 downstream of local steroid action in the endometrium of women with HMB during menstruation. Endometrium from women with objectively measured HMB had decreased TGF-*β*1 protein levels, unaltered TGF-*β* receptor presence, and a significant reduction in both SMAD2 and 3 mRNA concentrations and SMAD2/3 protein phosphorylation before/during the menstrual phase when compared with women with NMB. We provide mechanistic data supporting TGF-*β*1 protein activation by cortisol in endometrial cells, via TSP-1. In addition, our functional studies reveal that a suboptimal TGF-*β* response in the local endometrial environment may decrease postmenstrual repair of the stromal compartment and lead to heavy, prolonged menstrual bleeding (Fig. 7).

Previous studies have detailed that TGF-*β*1 levels in endometrial tissue explants are suppressed by progesterone (8). These authors found secretory explants cultured for 24 hours in the absence of progesterone and estrogen, a milieu analogous to the menstrual phase, significantly increased *TGFβ1* mRNA. Our results support these findings, with significantly greater *TGFβ1* mRNA prior to and during menstruation when compared with the proliferative and early-mid secretory phases, consistent with upregulation following progesterone withdrawal. We did not observe any significant difference in endometrial *TGFβ1* mRNA between women with HMB and normal blood loss during menstruation, although we acknowledge our n numbers are small. However, we did observe significantly decreased TGF-*β* protein in the stromal compartment of women with HMB *versus* NMB during menstruation. We acknowledge that menstrual biopsy n numbers are low, but these tissues are meticulously classified and have objective measurement of participant menstrual blood loss to aid precision of data. Our results suggest differences in TGF-*β*1 protein in women with HMB and NMB are not due to transcriptional regulation, but that posttranscriptional regulation may be aberrant.

Interestingly, there were no significant differences in serum progesterone or estradiol levels between women with HMB and NMB. In addition, no significant differences in endometrial estradiol receptor or progesterone receptor expression were previously detected in women with measured menstrual blood loss (18). Therefore, we hypothesized that local cortisol action may influence TGF-*β*1 activity during menses.

TGF-*β* is synthesized as a dimeric preproprotein and is released in a latent form. TSP-1 is known to activate TGF-*β*1 and is thought to do so by inducing a conformational change in the latent protein (6). Our laboratory has previously published that women with HMB have significantly reduced endometrial TSP-1 mRNA levels when compared with women with normal bleeding (14). Previous studies from our laboratory have also found that cortisol increases TSP-1 mRNA expression in primary human endometrial stromal cells (14). Direct measurement of cortisol levels in the endometrium of women with HMB and NMB has not yet been carried out, but an enhanced local inactivation of cortisol by 11*β*HSD2 may be present in the endometrium of women with heavy menses (14). The 11*β*HSD2 mRNA was increased 2.5-fold in women with HMB *versus* NMB, predicting substantially lower local cortisol concentrations. Therefore, we examined whether cortisol was a local regulator of TGF-*β*1 activity via TSP-1. On examination of cell culture supernatants from HES cells treated with physiological levels of cortisol (17), activated TGF-*β*1 was significantly increased. This increase was abrogated by the addition of a TSP-1 inhibitor to culture. Interestingly, acid activation of latent TGF-*β*1 in the culture supernatant prior to ELISA resulted in no differences in TGF-*β*1 levels with any of the treatments used. This is consistent with cortisol-stimulated TSP-1 production acting on latent TGF-*β*1 protein to increase its activity, rather than increasing the transcription or translation of TGF-*β*1. Indeed, cortisol and cortisol plus TSP-1 inhibitor treatment both significantly decreased *TGFβ1*. *TGFβ1* mRNA was not significantly different in the endometrium of women with NMB *versus* HMB during the perimenstrual phase, but there was a trend toward increased TGF-*β*1 mRNA concentrations in women with HMB at this time, consistent with lower endometrial cortisol levels (14).

Next, we examined the functional significance of TGF-*β*1 protein levels on endometrial cells. After shedding, endometrial cells migrate to cover the exposed surface of the endometrium and the stromal compartment regenerates (19). The wound scratch assay mimics this process *in vitro*, providing a means of quantifying stromal cell migration across a wounded surface. We found that TGF-*β*1 increased wound healing of primary stromal cell cultures. As we detected reduced phosphorylation of SMAD2/3 in the endometrium of women with HMB *versus* NMB, we blocked TGF-*β*–mediated activation of SMAD proteins with SB 431542 and showed a decrease in stromal cell wound migration, which was significantly less than that seen with the addition of TGF-*β*1. We propose that women with HMB may have defective or delayed repair of the stromal cell compartment following shedding of their functional endometrium at menses.

In addition to its functional role in proliferation, it is clear that the TGF-*β* superfamily plays an important role in endothelial cell function and blood loss. Greater than 50% of TGF-*β*1 knockout mice die during embryogenesis due to yolk sac defects affecting vasculogenesis and resulting in vessel fragility (20). In humans, mutation of the TGF-*β* receptor I activin receptor-like kinase I or of the endothelial accessory receptor endoglin causes hereditary hemorrhagic telangiectasia, an autosomal dominant vascular disease (21). The resulting aberrant TGF-*β* superfamily signaling results in epistaxis, telangiectasia, and arteriovenous malformations. Interestingly, previous histochemical and microscopic examination of endometrial blood vessels from women with normal and HMB revealed increased endothelial gaps in women with heavy loss (22). The role of the TGF-*β* superfamily in this pathology remains to be determined, but the observational data contained in this work suggest that low late secretory/menstrual TGF-*β*1 protein levels and decreased pSMAD2/3 may be involved. Previous results from our center support a role for TGF-*β*1 in the generation of vasoactive factors in women with endometriosis (23, 24) and it may have a similar, if more regulated, role in the endometrium to ensure physiological menstruation.

We have previously shown that cortisol is angiostatic, preventing endothelial tubelike structure formation *in vitro* (14). Furthermore, small interfering RNA silencing of TSP-1 in uterine endothelial cells reversed the antiangiogenic effect. In combination with data contained in this work, we propose that cortisol may activate endometrial TGF-*β*1 via TSP-1 during menses to prevent an excessive angiogenic response and increase vascular integrity. Further experiments are required to definitively test this hypothesis.

In conclusion, we show that women with objectively measured HMB have decreased endometrial TGF-*β*1 protein and downstream SMADs during the late secretory/menstrual phase when compared with women with NMB. This may partially explain the increased menstrual blood loss experienced by many women. In addition, we show that cortisol has a mechanistic role in the activation of endometrial TGF-*β*1 at this time (Fig. 7). Our *in vitro* results are consistent with TGF-*β*1 having a functional role in repair of the denuded endometrial surface at menstruation, and we propose that women with HMB may benefit from therapies that increase TGF-*β* during menses.
