# Supplementary material for: Reduced Transforming Growth Factor-β Activity in the Endometrium of Women With Heavy Menstrual Bleeding
Source: J Clin Endocrinol Metab. 2017 Jan 3;102(4):1299–308. doi: 10.1210/jc.2016-3437 (PMC5460733; doi:10.1210/jc.2016-3437)
Supplement: Supplementary file 1 [file jc.2016-3437.st1.pdf]

Supplementary Table 1. Serum Estradiol and Progesterone levels taken at time of endometrial biopsy.

Stage of Cycle	Blood loss	Estradiol pmol/l Mean (range)	Progesterone nmol/l Mean (range)
Proliferative	NMB (n=11)<br>HMB (n=10)	784 (178-1942)<br>574 (142–1657)	3.0 (1.0 – 6.6)<br>3.6 (1.1-10.1)
Early-Mid Secretory	NMB (n=15)<br>HMB (n=13)	396 (130-919)<br>450 (145-904)	41.4 (13.4-119.0)<br>47.7 (14.2-124.8)
Late Secretory	NMB (n=9)<br>HMB (n=8)	213 (90-373)<br>231 (70-368)	11.1 (9-14.1)<br>14.0 (9.6-18.9)
Menstrual	NMB (n=8)<br>HMB (n=4)	187 (85-392)<br>103 (50-144)	2.5 (1.6-5.1)<br>5.3 (1.5-18.4)

Supplementary Table 2. Immunohistochemistry Antibody Details

Antibody	Supplier	Concentration
Rabbit anti-human TGFβ1 (SC-146)	Santa Cruz	1:100
Rabbit anti-human TGFβ receptor 1 (SC-398)	Santa Cruz	1:2000
Rabbit anti-human TGFβ receptor 2 (SC-220)	Santa Cruz	1:400

Supplementary Table 3. Primer details

Gene	Accession number	Forward Primer	Reverse Primer
TGFB1	NM_000660.4	ACTACTACGCCAAGGAGGTCAC	TGCTTGAACTTGTCATAGATTTCG
TGFBR1	NM_004612	GGTCTTGCCCATCTTCACA	GTTCCCACTCTGTGGTTTGG
TGFBR2	NM_001024847	CTGGTGCTCTGGGAAATGAC	CGTCTCACACACCATCTGGA
SMAD2	DQ893746	CCAGGTCTCTTGATGGTCGT	GTCGGGGCACTAATACTGGA
SMAD3	NM_005902.3	CCATCCCCGAAAACACTAAC	TCCATCTTCACTCAGGTAGCC

Supplementary methods: Serum ovarian hormone assays

Oestradiol was performed on a Roche Cobas E411 immunoassay analyser (Roche Diagnostics, Burgess Hill, UK) according to manufacturer's instructions. The lower limit of detection is reported at 18.4 pmol/l. Within assay %CV was found to be <5% and between batch CV 6.5% and 5.7% for the low and high quality control material respectively.

Progesterone ELISA was performed by coating 96-well plates with 100µl of goat anti mouse IgG (Jackson ImmunoResearch Inc, West Grove, USA) per well at a dilution of 1:500 in ELISA coating buffer (100mM Na Bicarbonate, pH 9.6) at 4oC overnight. Standards, samples and controls (20µl per well) were added in duplicate, followed by 80µl of Progesterone 3 - HRP conjugate (Astra Biotech GmbH, Berlin , Germany) at 1:20,000 (PBS pH 7.4 containing 0.1 %BSA and 250 ng/ml Cortisol), followed by 50µl monoclonal progesterone Ab (Meridian Life Sciences, Memphis, USA) 1:100,000. Plates were incubated at room temperature for 2 hr then washed 5 times with assay wash buffer and 120µl of substrate solution (3,3,5,5-Tetramethylbenzidine, Millipore Corporation, Temecula, CA, USA) was added to each well. The reaction was stopped by adding 80µl of 2N H2SO4 solution (Sigma-Aldrich Company Ltd., Dorset, UK). Plates were read at 450nm. Standard curves were prepared (16, 8, 4, 2, 1, 0.5, 0.25 0 ng/ml). The inter-assay CV for low and high pools respectively were 11.4 and 9.1% the intra-assay CV were 8.9 and 5.6%. The lower limit of detection was 0.1 ng/ml. Cross-reaction: estrone: 0.17%, estradiol: 0.28%, estriol: 0.18%, dehydroepiandrosterone: 0.02%, testosterone: 0.36%, dihydrotestosterone: 0.15%, 17α-hydroxyprogesterone: 2.9%, androstenedione: 0.14%, 11-deoxycortisol: 0.46%, corticosterone: 0.18%, cortisone: 0.04% and cortisol: 0.04%.
